# Populations of Tau Conformers Drive Prion-like Strain Effects in Alzheimer’s Disease and Related Dementias

**DOI:** 10.3390/cells11192997

**Published:** 2022-09-26

**Authors:** Lenka Hromadkova, Mohammad Khursheed Siddiqi, He Liu, Jiri G. Safar

**Affiliations:** 1 Department of Pathology, Case Western Reserve University School of Medicine, Cleveland, OH 44106, USA; 2 Department of Nutrition, Case Western Reserve University School of Medicine, Cleveland, OH 44106, USA; 3 Department of Neurology, Case Western Reserve University School of Medicine, Cleveland, OH 44106, USA; 4 Department of Neuroscience, Case Western Reserve University School of Medicine, Cleveland, OH 44106, USA

**Keywords:** Alzheimer’s disease (AD), frontotemporal lobar degeneration (FTLD), misfolded tau protein conformers, prion-like tau strains

## Abstract

Recent findings of diverse populations of prion-like conformers of misfolded tau protein expand the prion concept to Alzheimer’s disease (AD) and monogenic frontotemporal lobar degeneration (FTLD)-MAPT P301L, and suggest that distinct strains of misfolded proteins drive the phenotypes and progression rates in many neurodegenerative diseases. Notable progress in the previous decades has generated many lines of proof arguing that yeast, fungal, and mammalian prions determine heritable as well as infectious traits. The extraordinary phenotypic diversity of human prion diseases arises from structurally distinct prion strains that target, at different progression speeds, variable brain structures and cells. Although human prion research presents beneficial lessons and methods to study the mechanism of strain diversity of protein-only pathogens, the fundamental molecular mechanism by which tau conformers are formed and replicate in diverse tauopathies is still poorly understood. In this review, we summarize up to date advances in identification of diverse tau conformers through biophysical and cellular experimental paradigms, and the impact of heterogeneity of pathological tau strains on personalized structure- and strain-specific therapeutic approaches in major tauopathies.

## 1. Phenotypic Spectra of Alzheimer’s Disease (AD) and Frontotemporal Lobar Degeneration (FTLD)

Alzheimer’s disease (AD) and Frontotemporal Lobar Degeneration (FTLD) are the prominent causes of age-related dementia [1,2,3,4,5]. Although AD is a dual proteinopathy with concurrent deposition of misfolded amyloid beta fragment and hyperphosphorylated tau aggregates, tau deposits are the best indicator of cognitive decline [6,7]. AD encompasses extraordinarily variable phenotypes and progression rates, categorized by the prevalent clinical symptomatology as an amnestic variant, posterior cortical atrophy (PCA), logopenic primary progressive aphasia (LPPA), and the frontal variant of AD [1,6,8]. Additionally, based on the neuroimaging and neuropathology data, these clinical phenotypes have been divided into typical AD, with balanced neurofibrillary tangle (NFT) depositions in the hippocampus and association cortex; limbic-predominant AD, with NFTs predominantly occurring in the hippocampus; and hippocampal-sparing AD, with counts mostly observed in the association cortex [7,9]. Moreover, approximately 10% of AD cases have a rapidly progressive form (rpAD) with an accelerated tempo of clinical decline and disease duration frequently of less than three years [10,11,12,13,14]. The sources of this clinicopathological heterogeneity including variable involvement of astroglia [15,16,17,18] are not fully understood and only ~30% of the variability can be attributed to genetic polymorphisms [19,20,21,22]. Immunohistochemistry data show that aggregated and hyperphosphorylated tau inclusions are present in glial cells in AD, FTLD-17, and other tauopathies [23,24], and in mice models as well [25,26,27], but how the intracellular trafficking in glial cells would impact propagation of different prion-like strains of tau aggregates is not known and different glia may have protective or enhancing effects [28,29,30].

Frontotemporal lobar degeneration (FTLD) is also a clinically, genetically, and pathologically diverse group of disorders and the umbrella term FTLD [31,32,33] can be divided into the behavioral variant (bvFTD) and (at least) two types of primary progressive aphasia (PPA): non-fluent variant (nfvPPA) and semantic variant (svPPA) [34]. The bvFTD accounts for nearly 60% of cases of FTLD; the language/semantic variants are less common. Intriguingly, the clinicopathological heterogeneity in the spectrum of sporadic FTD-tau disorders is also well documented in their genetic forms such as FTLD-MAPT-P301L, with a diversity of clinical manifestations observed among patients carrying the exact same mutation, and even in the same family [32,35,36].

The distribution of tau pathology in both AD and FTD-tau tends to correlate with cognitive decline, brain areas of atrophy, and the disease stage, and therefore tau misfolding and aggregation is considered to be a link to common downstream mechanisms of neurodegeneration [3,6,16,28,36,37]. Comprehensive analysis of aging brain samples indicates that the pathological changes linked to AD start early on, through the accumulation of misfolded and hyperphosphorylated tau species in isolated anatomical areas of the brain, which then spread by cell-to-cell transmission mechanisms [17,38]. The increasing evidence from cell and transgenic mice models show that various misfolded and aggregated tau strains generated in vitro or in vivo can replicate in cells [39], hasten and spread endogenous tau aggregation in recipient transgenic animals [40,41], and therefore indicate a prion-like replication phenomenon. Although aggregates of other proteins linked to a growing number of neurodegenerative diseases can be propagated from human tissues to experimental animal models or between humans [42,43], there is currently no evidence for direct infectious etiology for the human neurodegenerative disorders defined by these proteins [44]. Because of the importance of this question, the mechanism of replication and propagation of misfolded proteins deserves primary attention, and improved understanding has important implications for individualized diagnostics and therapeutic strategies.

## 2. Implications of Strain Concept of Human Prion Diversity for Tauopathies

Human prions cause perhaps the most heterogenous neurodegenerative disorders, which were until recently considered separate diseases; the classification was based on leading clinical symptoms or they were named after the author(s) who originally described them—sporadic and familial fatal insomnia (sFI, FFI), Creutzfeldt–Jakob disease (CJD), Gerstmann–Sträussler–Scheinker (GSS) disease, variably protease-sensitive prionopathy (VPSPR), and a spectrum of unclassified rare genetic forms [45,46,47,48,49]. Human prions are transmissible from person to person [43], to primates [50,51], and to experimental animals [52,53], and the existence of distinct prion phenotypes in the host expressing the same prion protein sequence was offered as an argument for the presence of a prion-specific nucleic acid [54,55]. However, even with frequent attempts to detect such a nucleic acid applying various approaches, and despite accumulating data contradicting the existence of a prion strain-coding polynucleotide [56,57,58,59], the existence of distinct prion strains remains a conundrum, and the most important challenge to fundamental principles of molecular biology [52,60,61]. Many lines of evidence now indicate that the extraordinary phenotypic diversity of human prion diseases arises from the structurally distinct prion strains that target variable brain structures at different progression speeds [45,46,47,48,62,63,64]. This paradigm is supported by biochemical, genetic, and animal studies [65,66,67,68,69], and lastly by a recent successful generation of the first synthetic human prions [70]. Expanding the principles developed with cloned laboratory prions, the general consensus criteria for human prion strains based on their clinicopathological and molecular characteristics are now used for differentiation, classification, and surveillance of human prion diseases in prion centers in the US and Europe (Table 1) [48,71,72,73].

## 3. Isoforms and Cellular Functions of Normal Human Tau Protein

Although genetic polymorphisms in APOE and a growing number of other genes are important risk factors of late onset AD, at least two features of late onset AD that cannot be explained by linkage analysis are (i) divergences between amyloid beta and tau pathological deposits and severity of clinical disease-specific manifestations [10,13,87], and (ii) the general variability of progression rates and phenotypes [6,88,89]. Early pivotal data generated with HEK reporter cells showed different patterns of tau aggregates after inoculation by tau isolates from distinct tauopathies [39] and recently even with different tau strains generated from monomeric tau in the absence of aggregated seeds [90,91]. These experiments inferred alternatively structured tau species encoding the key information for distinct phenotypes seen in cell experiments with prion-like mechanisms. However, the aggregates of fluorescently labeled tau accumulating in HEK reporter cells in response to the inoculation with conformationally different strains do not correspond to the AD tau filament assemblies [92], but rather show various morphologies and intracellular locations. These cell patterns suggest a seeding barrier effect due to the presence of mutation in the HEK cell-expressed short K18 tau fragment.

**Figure 1:**
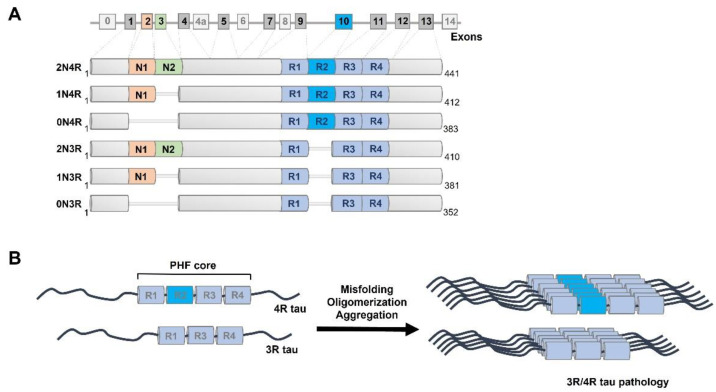
Alternative splicing of MAPT gene and tau protein aggregation. (**A**) Alternative splicing of exons 2, 3, and 10 leads to six isoforms of tau in the adult human brain; exons 4A, 6, and 8 are exclusive to the peripheral nervous system and absent in the human brain [104]. Six isoforms of human tau occurring in the central nervous system (2N4R, 1N4R, 0N4R, 2N3R, 1N3R, 0N3R) vary by the presence or absence of N-terminal regions (N1, N2) and repeat domain 2 (R2), resulting in molecular weights ranging from 36,760 Da (0N3R) to 45,850 Da (2N4R). (**B**) Tau is under physiological conditions an intrinsically disordered protein that dynamically interacts with and stabilizes microtubules. Under pathological conditions, tau repeat region cores undergo conformational transition to beta sheet-rich secondary structures prone to formation of oligomers and paired helical filaments (PHF) [105] with amyloid tinctorial properties.

In contrast to the human prion protein (PRNP) gene that has a single open reading frame [47,48], the MAPT gene undergoes complex alternative splicing in the coding region (Figure 1). The MAPT gene is located on chromosome 17q21 and possesses 16 exons in the tau primary transcript. Unlike exons 1, 4, 5, 7, 9, 11, 12, and 13 that are constitutive, exons 2, 3, 4a, 6, 8, and 10 are subject to alternative splicing [93] (Figure 1). Overall, alternative splicing results in six tau isoforms that differ by the presence or absence of two N-terminal regions (0 N, 1 N, or 2 N) as well as conserved repeat motifs (3R or 4R) [94,95]. In the brain, tau mRNA is expressed primarily in neurons, but low expression levels of tau can be found in various types of glial cells, including astrocytes and oligodendrocytes [15,96,97]. The main function of tau as a member of the microtubule-associated proteins (MAPs) group is to bind and stabilize microtubules through the microtubule-binding domain located at the C-terminus of tau, mediate their assembly, and thus modulate vesicle/organelle transport on microtubules [98,99,100]. The N-terminal domains may influence spacing between microtubules, subcellular distribution of neuronal tau, and aggregation kinetics of tau [101,102]. The repeat motifs constitute the microtubule-binding domain of tau, and the proline-rich domain (PRD) links the C-terminal assembly domain and the N-terminal projection domain. Overall, due to its high content of polar and positively charged amino acids, tau is a water-soluble protein with a high isoelectric point. Monomeric tau lacks a stable secondary and tertiary structure and instead possesses great structural flexibility [103].

Neuronal tau is principally located in the axons and to a lesser degree in somatodendritic compartments such as the cell membrane [106], mitochondria [107], and nucleus [108]. Apart from important roles of tau in axonal transport [109,110], it also plays a role in nucleic acid protection [111], synaptic plasticity, and neuronal maturation [112,113,114]. Structural studies confirm that unbound tau is indeed an intrinsically disordered protein (IDP) [103] that possesses the tendency to change its unstable random conformations to more energetically favored, detergent-insoluble, partially protease-resistant protein aggregates [115] with amyloid-like tinctorial properties and high affinity for amyloid dyes-Thioflavin S, Congo Red, and their derivatives [116,117] (Figure 1). This dramatic structural transformation leads to the loss-of-function and gain-of-function outcomes that are a critical step in the pathogenesis of AD and FTLD, and in general all tau-related neurodegenerative diseases-tauopathies.

The sequence alterations in tau microtubule-binding domains that occur in mutations associated with FTLD-17 cases lead to reduced interaction with microtubules, altered ratio of 3R and 4R isoforms in favor of 4R tau, and misfolding of tau protein [118,119,120,121]. Numerous transgenic mice lines expressing mutant forms of human full-length tau were developed to investigate tau pathology at an accelerated rate. In parallel, to address the overexpression of tau linked to several mutations identified in FTDP-17 cases [120,121], the ALZ17 mice line expressing the non-mutated longest human tau 4R isoform was introduced [122]. The missense mutations such as P301L/S, W337M, and R406W result in reduced ability of tau protein to bind with microtubules and accelerated misfolding and aggregation [119,121]. These well established and extensively characterized [123,124,125,126,127,128,129,130,131,132,133] transgenic systems provide valuable tools not only for studying tau pathology caused by these mutations but also animal models for monitoring tau seeding potency and propagation of tau aggregates after intracerebral injection [132,133,134,135]. The expansion of mice models expressing various mutated/non-mutated tau isoforms has an enormous impact on understanding tau pathology initiation and spread, and several excellent reviews recently summarized the progress in this field [136,137,138,139,140].

## 4. Preferential Misfolding of 4R Tau Isoform in Late Onset AD and FTLD-MAPT-P301L Patients

**Figure 2:**
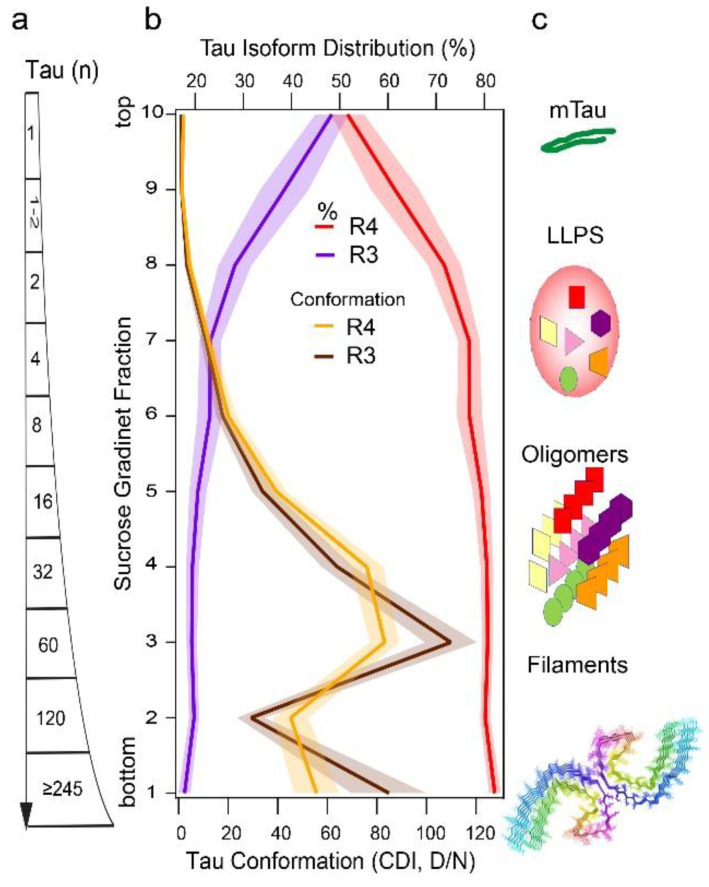
Size, 3R and 4R isoform distribution, and conformation of tau particles in the hippocampus of AD cases. (**a**,**b**) Size, concentration, and conformation of insoluble misfolded 4R and 3R tau fractionated by sedimentation velocity using ultracentrifugation in sucrose gradient were analyzed simultaneously by 4R- and 3R-specific CDI. (**c**) The schematic representation of normal tau monomer and different structural forms of misfolded tau proteins-induced ensembles of dynamic liquid-liquid phase separation (LLPS) [146], oligomers [147], and PHF structures [105]. The composite sucrose gradient is replotted from recently published data [10] and the structure of PHF was generated by Cn3D application from published coordinates [105].

The expression of all six tau isoforms in the adult human brain led to the assumption that neurofibrillary tangles (NFTs) are a result of random integration of different tau isoforms into paired helical filaments (PHFs) and straight filaments (SFs) in AD [141,142,143,144]. However, the sedimentation velocity separations of total brain homogenates by ultracentrifugation in sucrose gradient show that misfolded tau aggregates are, regardless of size, uniformly composed of ~80% of 4R tau and ~20% of 3R tau, even though the soluble normal tau monomers are mixtures of approximately equal proportions of 3R and 4R isoforms [10] (Figure 2). This altered distribution of 3R and 4R tau with 4-fold excess of 4R in misfolded tau aggregates indicates higher susceptibility of unfolded 4R tau monomers to conformationally convert to beta-sheet structures in AD, which correlates with seeding experiments using K18 and K19 tau constructs. Thus, 4R aggregates are likely formed with faster conversion kinetics and more rapid growth might be the mechanism explaining the 3R, 4R unequal distribution in AD tau aggregates [10]. However, whether the distinct individual tau aggregates are composed of only 4R and 3R or by both isoforms has been under debate. The recent cryoEM models of core PHFs extracted from AD brains may accommodate upstream of V306 an additional 16 amino acids, which could represent a mixture of residues 259–274 (R1) from 3R tau, or 290–305 (R2) from 4R tau [143,144], but in contrast, show 1:1 stoichiometry of R4 and R3 tau isoforms. Latest reports from Scheres’ group indicate that in vitro forming filaments of misfolded tau are exceptionally conformationally flexible and might consist of a pool of exceedingly unstable conformers with numerous structural transitions dependent on ambient conditions [104], but how to translate these in vitro experiments to aggregated and misfolded human tau samples might be mostly limited by the application of standard purification procedures. Using cell biosensors, several groups recently showed that the seeding capacity of tau present in various AD brain patients’ samples precedes the NFT formation and shows high variability between individual samples [10,145]. Cumulatively, the data imply (i) the existence of separate pathways for PHF and SFs, and (ii) large pools of misfolded tau with nonfibrillar morphology of the aggregates observed in ultracentrifugation sedimentation velocity experiments [10]. The different sizes of misfolded tau particles separated in sucrose gradients with distinct conformations corroborate this conclusion and implicate liquid-liquid phase separation (LLPS) and oligomeric states of tau (Figure 2). Notably, conformation of 3R tau in tau aggregates monitored by CDI and CSA resemble conformational profiles nearly identical to those mixed of (3R + 4R) tau [10]. To better understand the single mixed particle versus distinct particle model in nonfibrillar aggregates will require advanced techniques such as immune(cryo)EM and hydrogen exchange mass spectrometry (HX MS).

## 5. Conformational Diversity of Tau Isoforms in Different Phenotypes of AD, FTLD-MAPT-P301L, and TgP301L Model

**Figure 3:**
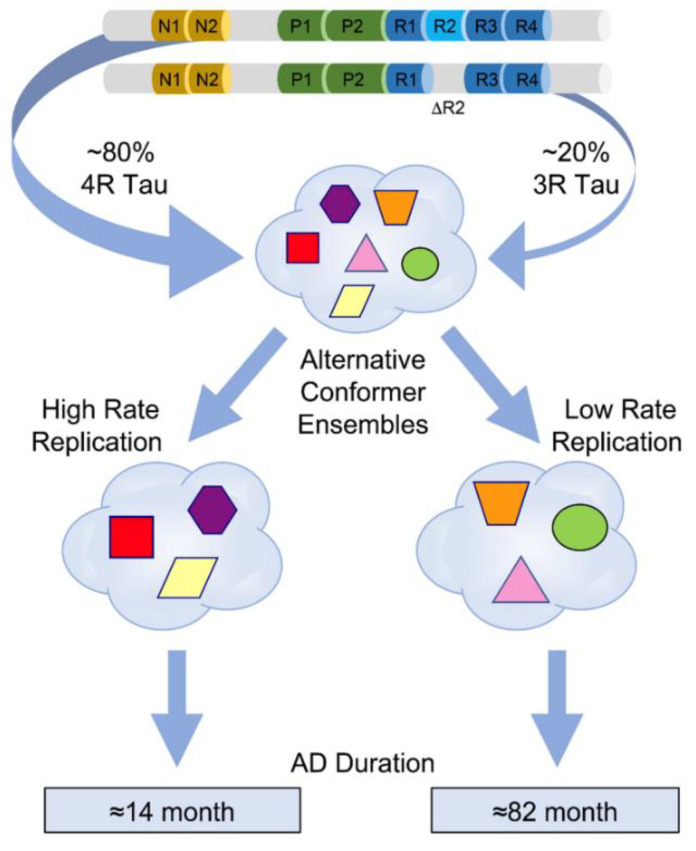
Evolving ensembles of misfolded tau conformers in the pathogenesis of different progression rates of Alzheimer’s disease. Different geometric shapes and colors represent predominantly four-repeat conformers of detergent-insoluble misfolded tau. Different coexisting combinations (ensembles) of conformers corresponding to different CSA profiles are shown within the cloud outlines. The data were obtained by deconvolution of conformational stability (CSA) types seen in 43 AD cases with variable progression rates and they likely represent alternative pathways of ensemble evolution (blue arrows). Figure is reprinted from [10] with permission of the American Association for the Advancement of Science, 2022.

The biophysical techniques that were originally developed for prions are ideally suitable to monitor the structural pattern and composition of tau directly in the brain tissue of AD cases with distinct phenotypes [46,84,85,86]. The main advantages of using these ultrasensitive methods to investigate conformational organization and properties of misfolded tau aggregates are no requirement for tau purification or in vitro amplification step before detection; they are independent of the absolute concentrations of misfolded protein particles, and thus the original characteristics of strain isolates are preserved [70,148]. While the indirect properties such as the size and protease resistance of tau aggregates and cellular or neuropathology patterns in the reporter HEK293 cells and transgenic mice [149], and posttranslational modifications (PTMs) of tau in mass spectrometry [8,147] suggested similar structure of tau in individual AD cases, the new direct biophysical and conformational data revealed evolving conformer populations driving in prion-like manner different phenotypes in individual AD [10] cases (Figure 3). This conformational paradigm is supported by the diversity of sedimentation profiles of tau aggregates (Figure 2), trypsin peptide maps, conformation-dependent immunoassay (CDI), and conformational stability assay (CSA) [10] and mirrors the data obtained with the phenotypic spectrum of FTLD-MAPT(P301L) cases and in the transgenic model of FTLD [150]. Additionally, recent advances in cryoEM provide unequivocal evidence for up to 76 distinct tau filament structures that can be generated in vitro with subtle changes in assembly conditions [104]. Cumulatively, these new findings implicate major conformational flexibility of tau in prion-like strain effects and the new hydroxyl radical footprinting approaches [76,151] to the tau strain investigation should provide unbiased structural information on tau conformers in the ensemble of all their misfolded states, particularly prefibrillar (oligomeric) forms and liquid-liquid phase separation (LLPS) droplets, and “fuzzy” coat in filamentous structures [112,146] (Figure 2).

The deposits of hippocampal tau are considered critical in cognitive decline because they are at the crossroad in the spread of pathogenic tau aggregates, from early deposits in transentorhinal cortex (Braak stages I–II) to major projections to the hippocampus where tau pathology gradually occurs in the CA1 region (Braak II), followed by spread to the limbic structures, inferior temporal neocortex (Braak III), the amygdala and thalamus (Braak IV), and finally propagation into the neocortex (Braak V–VI) [38,40]. Notably, another predominantly 4R tau tauopathy-frontotemporal lobar degeneration (FTLD)-MAPT-P301L-also displays three distinct tau signatures in cases with different clinical phenotypes, two resembling those found in prodromal TgTauP301L mice model of FTLD [150]. As biophysical methods and HEK293 cell biosensors confirm a broad spectrum of tau strains in the mouse and human brain series, the evolution of diverse tau conformers is apparently an intrinsic feature of both tauopathies’ pathogenesis-uniallelic form of FTLD [150] and AD [10].

Another important aspect contributing to the structural complexity of tau aggregates are posttranslational modifications (PTMs). In recent studies, the correlation between seeding potency and various PTM profiles obtained by mass spectrometry of misfolded insoluble tau isolated from different clinical phenotypes has been reported [147,152]. Similar trends are also being observed at the early age of onset in familial Alzheimer’s disease (FAD) with Presenilin-1 (PSEN1) mutations [8]. These observations suggest a closer relationship between posttranslational modification and structural organization of tau during AD progression and raise the question of whether the variances in conformations are driven by distinct PTMs or vice versa. Even though the tau phosphorylation at specific sites is associated with aggregated tau burdens and is used in diagnostic and immunohistological confirmation of tauopathies [153,154,155], the causative effect of PTMs on tau misfolding and generating tau seeds competent to spread the tau pathology is debated in light of recent data that show seeding activity even in the absence of phosphorylated tau deposits [40,156,157,158]. These are important effects not only in AD and FTLD, but in other tauopathies as well, including progressive supranuclear palsy (PSP), and Pick’s disease. The interplay between PTMs and the formation of misfolded structural orders of tau may be simultaneously investigated by using high advanced approaches such as integrated cryoEM combined with conformation-sensitive mass spectrometry (HX MS) and/or synchrotron footprinting, which are applicable for both fibrillar and oligomeric forms of misfolded tau [46,70,76,159]. These approaches should also determine whether the sedimentation velocity and different trypsin peptide patterns of conformers separated by sedimentation velocity are exclusively a result of conformation and particle size, or are modified by posttranslational modifications, or Sarkosyl-resistant ligands which could be important cofactors in the tau misfolding.

## 6. Modelling Replication Mechanism of Tau Conformer Populations In Vitro

The important evidence for replication and propagation of misfolded tau conformers in the brain is that the misfolded protein can be amplified in vitro in a seeded reaction [70,160]; this concept was proved first with laboratory prions [148], later with human prions [71], and more recently with an increasing number of other misfolded prion-like proteins including alpha synuclein [161] and TDP-43 protein [162]. The conformational templating process for amplification of AD brain-derived tau seeds utilizes purified recombinant K18 (4R) and K19 (3R) tau substrates or their chimeras [163,164], and at the same substrate concentrations, the AD brain-derived tau seeds preferentially amplify with K18 (4R) substrate and demonstrate end point sensitivity up to 10^9^ dilution of the brain tissue [10,165,166]. Using the tau assay, recent reports are consistently showing major interindividual variability of seeding potency of AD brain-derived tau [160,165,166] and homogenates of rapidly progressing AD cases demonstrate a significantly shorter lag phase than classical slowly progressing cases [10]. Cumulatively, these data show that in individual AD cases there is a cloud of distinct conformers of misfolded tau with various seeding potencies and that the better and more reliable predictor of seeding potency is conformation of insoluble 4R tau, which correlates with faster disease progression, than the absolute concentration of all misfolded tau seeds.

The ultracentrifugation in sucrose gradient [10], chromatographic separations [147], protease sensitivity [10], oligomer-specific antibodies [167], and lower stability of some of misfolded tau structures in conformational stability assays [10] all indicate that a significant portion of misfolded tau is in an oligomeric or prefibrillar state (Figure 2). The systematic examination of three different human prion strains has revealed that high affinity of surface domains in strain-specific prion conformers for their substrate (PrPC) results in faster replication and propagation of human prions [46,70,76]. The new biophysical data on AD tau are in agreement with this concept and point to a conformational cloud of distinct tau conformers with different interactions between misfolded and monomeric tau and affinity-driven faster replication in rapidly progressive AD [10]. The differences in these populations found in rapidly and slowly progressing AD are linked to the different replication rates in vitro in seeding assays and in cell reporters [10]. The mechanisms or aspects behind these differences are not fully understood, but the assembly of quaternary oligomer or fibrillar structures and the structure of monomeric building blocks must be thermodynamically and kinetically linked [168]. Consequently, the differences in replication slopes seen in in vitro seeding assays point to distinct mechanisms such as fibril growth from ends or from sites as theoretically suggested [169]. Additionally, the studies of prion strains of both human and animal origin imply that non-protein cofactors (phospholipids, gangliosides, negatively charged polyanions, etc.) might contribute to the diversity of misfolded strains by affecting their stability and propagation [70,159,170,171,172]. Such auxiliary cofactors in tau strains remain to be found, but they could contribute to our understanding of propagation rates of specific tau strains and their affinity to differential interactomes and distinct host cells.

## 7. Strain Effects of Prion-like Tau Conformers in Cell Reporters

The substitution of proline 301 with lysine (P301L) or serine (P301S) in MAPT gene is the most common mutation associated with hereditary frontotemporal dementia with parkinsonism-17, FTDP-17 [32]. This single mutation facilitates fibril formation [173,174,175] and enhances seed recruitment in cells overexpressing human mutant tau variants [40,41,133,176,177,178,179,180,181,182,183]. The recruitment of wild-type tau by P301L tau fibrils was less efficient in non-transgenic primary neurons than in P19 mice neurons [133], and an even more evident asymmetric cross-seeding barrier was repeatedly observed in other in vitro studies [184,185]. The proline substitution in tau has multiple impacts: (i) The P301L mutant has higher propensity for assembly into fibrils [134]; thus the faster incorporation of mutated tau monomers into fibrils is preferential and it was shown that tau recruitment is a time-dependent event [133]. (ii) P301L mutant tau filaments are conformationally distinct from wild-type tau filaments [185,186]. (iii) Proline 301 is located at R2, which is encoded by exon 10 and present only in 4R tau isoforms; thus this single amino acid substitution might also lead to a different ratio of tau isoforms recruitment into filamentous inclusions [33,186]. (iv) The post-translational modifications may vary between propagated as well as templated seeds of P301L-tau and wt-tau, and thus contribute to misfolding [156].

Immortalized cell lines expressing full-length tau or its truncated variants with the most frequent disease-related mutations allow the monitoring of intracellular templated amplification of misfolded tau seeds. A number of cell lines are experimentally used for this goal, namely N2a [187], HEK293 [10,39,146,177,178,188,189,190], HeLa [191], and SH-SY5Y [192]. The most frequently used are HEK293 cells that were first introduced to prion research in experiments with GFP fusion proteins of yeast Sup35p prion substrate [193]. The experiments with different tau seeds performed in vitro showed that variable aggregate morphologies can be vastly transmitted in a clonal fashion in HEK293 cell stably transfected tau protein fragments (with P301L and V337M missense mutations) fused to a YFP reporter [39,177]. The subclone (Clone 1/DS1) of these reporter cells shows various morphologies of diffuse and amorphous shapes, nuclear envelope inclusions, speckles, and threads after inoculation with conformationally different strains of tau isolated human FTLD samples and Tg mice model of FTLD(P301L) [150].

Brain extracts from transgenic mice with distinct conformational organizations of tau demonstrated considerably different seeding profiles. Interestingly, tau-enriched extracts from human brains of patients with different clinical diagnoses (behavioral variant FTLDs with/without predominant memory impairment and semantic variant of primary progressive aphasia) displayed less pronounced differences in seeding profiles using by this assay. In analogous experiments, all samples of brain-derived AD tau induce tau aggregates with speckled morphology, but notably elevated prion-like seeding activities and higher frequency of positive cells exposed at the same concentrations of tau extracted from malignant AD with rapid progression [10].

The in vitro experiments indicate an early rapid conformational transition of intracellular tau upon seeding with brain-derived FTLD tau (P301) and formation of dispersed fluorescence signals that may next be collected and relocated within processes between nearby cells [146]. The fluorescence signal of tau within a nuclear envelope and small fluorescent inclusions has all the characteristics of liquid-liquid phase separation (LLPS)—Thioflavin S signal, spherical morphology, fusion events, and may recover from photobleaching [146]. These juxtanuclear tau assemblies are apparently linked to disrupted nuclear transport and decreased cell viability. These conformation-specific and dynamic cellular effects likely represent early steps in the transition to prefibrillar (oligomeric) and fibrillar tau inclusions seen in end-stage tauopathies. These observations also suggest that using immortalized cells to perform seeding experiments, the impact of cell cycle has to be considered as distinct tau inclusion types may accumulate in different stages of the cell cycle [10,112].

## 8. Effects of Misfolded Tau in Neuronal Cultures

Immortalized cell lines offer fast and high throughput tools to investigate the misfolded tau seeding in a cellular environment that overexpresses tau protein; but experimental factors such as genotype of recipient cells, expression of tau fragments with P301L and V337M missense mutations (which increase the tendency to aggregate and may introduce “species barrier”-like effects due to the tau sequence mismatch), acting together, may impact the outcome and thus deserve careful consideration. Thus, using neuronal cultures is a critical next step to enhance our knowledge about the interactomes and mechanism of replication of different tau conformers. Numerous factors have to be considered in the investigation of cell-to-cell tau aggregation transmission and cell-to-cell spreading in non-overexpressed tau cell systems: (i) origin, character, and concentration of pathological tau aggregates; (ii) uptake of tau aggregates without artificial cationic lipid-mediated transfection-presence of receptors like heparan sulfate proteoglycans and LRP1 [179,194]; (iii) character of tau in recipient cells-species origin, ratio and distribution of tau isoforms; and (iv) maturation of cultures, neuronal differentiation, and cellular network to sufficiently spread tau aggregation among cells.

**Figure 4:**
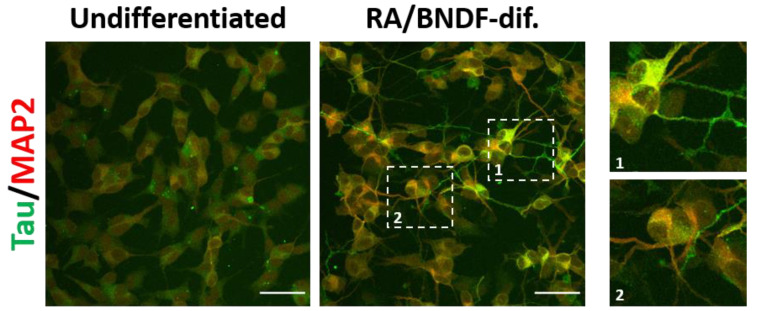
Tau and MAP2 expression and localization in undifferentiated and fully (RA/BDNF) differentiated SH-SY5Y cells (cell source: ECACC). The RA/BDNF differentiation facilitates the enrichment of both microtube-associated proteins, tau and MAP2, respectively. Moreover, two distinct types of neuronal projections, associated with a polarized state of neurons, are observed in fully differentiated SH-SY5Y: axonal-like neurites enriched with tau protein and somato-dendritic compartment consisting of MAP2 (cropped images 1 and 2). Scale bars: 50 μm. Figure is modified with from [197] with permission of Elsevier, 2022.

Human SH-SY5Y neuroblastoma, mouse N2a neuroblastoma, and catecholaminergic CAD cells among others are immortal cell lines with potential to be differentiated into neuronal subtypes expressing endogenous tau [195,196,197] (Figure 4). Overexpression of tau variants in these cells have been applied in most studies of tau pathology [187,198,199]. With the advantage of human origin, SH-SY5Y differentiate into polarized neuron-like cells with axonal sorting of human endogenous tau with both 3R, 4R isoforms [200,201] and have served as an alternative sporadic cell model for studies of pathological tau uptake, transmission and spreading [202,203].

Rodent primary neurons have been the gold standard cultures to study properties and behavior of post-mitotic fully polarized neurons with developed synaptic networking. In particular, neurons from non-transgenic mice initially express both 3R and 4R isoforms [41,204], but culture aging shifts the expression towards 4R isoforms, and thus they serve as a valuable cell model to investigate the tau misfolding propagation under conditions more resembling sporadic tauopathies. This is particularly important for investigating the cross-seeding barrier of 3R and 4R tau isoforms, which has been observed in vitro and in both reporter cells systems overexpressing tau [198,205] and primary neurons [41,206]. Overall, in vitro generated seeds from recombinant tau have a slower rate in extension of filament formation in cells than naïve tau fibrils isolated from brain material in in vitro cultures [41,181], and the distinct conformation of heparin-induced filaments and those isolated from AD brains was recently confirmed by cryo-EM studies [141]. The critical denominator of cellular effects is the structural organization of tau strains, including the size of aggregates [181,207] and only a small number of filaments with high seeding activity can trigger monomeric tau misfolding [41,176]. Several studies have shown that naïve fibrils from clinicopathologically different tauopathies have specific properties and their biological activity is likely to be associated with unique isoform composition and distinct conformational variants [206,208,209].

Over the past two decades, induced pluripotent cells (iPSCs)-derived neurons have become a valuable tool to investigate mechanisms of many neurodegenerative diseases [210,211]. There are almost limitless sources of healthy donors and patient-derived neurons that can be applied to uncover disease-specific alterations and serve as a platform to identify novel therapeutics via high throughput drug screening. Human iPSC-derived neurons grow and mature in cultures from weeks to several months and express predominantly fetal 0N3R tau isoform at early culture time-points, and only slightly shift expression to 3R, 4R representation during differentiation and maturation [212,213,214]. Importantly, neuronal progenitor cells are easily transfected with full length tau variants before differentiation including 2N4R-huTAU-P301L construct and full-length 4R tau constructs [215,216]. Although the presence of P301L mutation facilitates aggregation via in vitro generated K18-P301L fibrils, it also introduces the cross-seeding barrier between non-mutated tau and P301L variants [215,216]. Data from hiPSC-derived neurons with 3R wild-type tau and bearing P301S mutation show the cross-seeding barrier. 2N4R P301L fibrils induce robust aggregation at P301S tau hiPSC-derived neurons but not in hiPSC-derived neurons with 3R wild-type tau. However, AD brain-derived tau aggregates caused more tau aggregation in wild type hiPSC-derived neurons than in hiPSC-derived neurons with P301S mutation [217]. In a recent study of non-transfected hiPSC-derived neurons from healthy donors, full-length tau oligomers were internalized and recruited endogenous tau to induce pathological aggregation [218]. To sum up, experimental data of transmission and spreading tau pathology in neuronal cultures depends on many factors with emphasis on the origin of tau seeds and tau protein expressed in recipient neuronal cells.

## 9. Concluding Remarks and Future Investigations

The clinical manifestations and disease phenotypes of both sporadic AD and monogenic FTLD vary significantly among individual patients, but the mechanisms responsible for the broad spectrum of disease phenotypes have not been fully understood. One of the main factors affecting variable pathogenesis and disease progression in tauopathies seems to be related to the conformational and structural heterogeneity of misfolded tau aggregates, which show distinct behavioral and intrinsic properties such as different propagation rates, targeting various cell types, toxicity effects, and interactomes. The central role of amyloid beta due to mutations in genes of amyloid beta precursor protein APP and its endoproteases was unarguably recognized in the pathogenesis of early onset AD; however, the missing strong correlation between amyloid senile plaque deposits and severity of sporadic AD [87], and failure of attempts to therapeutically target amyloid beta-associated pathology in AD have opened more space to investigate tau protein involvement in AD [6,219]. The burden of tau aggregates, the second invariable marker of AD, correlates with cognitive decline and thus indicates the important role of tau pathology progression especially in sporadic forms of AD and FTLD. To improve our understanding of tau protein, its structural plasticity and its various misfolded and aggregated forms is the next critical direction for elucidating the disease mechanisms, and thus new targets for therapeutic interventions.

The structural evolution of tau pathological species in the familiar form of FTLD linked to MAPT P301L mutation [150] and recently published data obtained by sensitive biophysical methods reveal a broad conformational range of distinct tau conformers that accompany different phenotypes and also various progression rates in AD. Moreover, the discovery of various strains of amyloid beta that are analogous to numerous distinctive clouds of tau conformers in rpAD [11,13,220] suggest a pathogenetic association and indicate the possibility of a dual prion-like synergy, as recently suggested from experiments in vitro [221]. Additionally, the polymorphisms of the APOE gene linked to AD cases and distinct tau strains propose a imaginable parallel to circumstances noticed with human prions, where the interplay between common polymorphisms in the prion protein gene (PRNP) and variable conformational characteristics of the pathogenic prion protein leads to vastly different disease clinicopathological outcomes [47,48,64]. These observations are crucial towards more individual-based diagnostics and more precise and beneficial molecular classification of AD subtypes as well as for better understanding of mechanisms contributing to the disease progression; they thus provide essential information for exploring further AD therapeutic approaches including the necessity to consider clouds of tau misfolded conformers with extensive interindividual variability. The prion-like strain effects of misfolded tau conformers lead to a series of critical mechanistic questions relating to the role of misfolded tau aggregates in distinct phenotypes of AD and FTLD: (i) What is the molecular mechanism of replication and propagation of distinct brain-derived pathogenic aggregates, particularly prefibrillar forms of tau, in different phenotypes of AD and FTLD-tau? (ii) Which structural domains of tau control the propagation, toxicity, and evident range of pathological and clinical features in AD and FTD? (iii) What is the impact of distinct posttranslational modification on the structure of tau in different strains? (iv) What is the replication potency of different tau conformers? Answering these questions is of fundamental importance for verifying and advancing the emerging concept pointing to structurally distinct prion-like strains [89,102] of tau as a critical differentiating factor in AD and FTLD-tau development. The early data are consistent with the possibility that patients with tauopathy may have distinct molecular drivers of clinical phenotypes and emphasizes the need for personalized structure- and strain-specific therapeutic approaches.

Biosensor reporter cell lines overexpressing tau mutant variants are continuing to be an invaluable tool in investigating the capacity and diversity of various “tau seeds” to aggregate the reporter tau and have generated valuable data. However, to understand the mechanisms of tau uptake, seed-templated propagation of endogenous tau aggregation, tau pathology propagation and transmission between individual neuronal cells and alterations in molecular pathways, differentiated neuronal-like cell lines, and particularly hiPSC-derived neurons will play an irreplaceable role in improving our understanding of the origin and pathogenesis of sporadic tauopathies.

## Figures and Tables

**Table 1:** Classification criteria of human prion strains.

Classification Criteria of Human Prion Strains	References
Species of human prion strain are determined by the amino acid sequence and polymorphism of the misfolded conformer (PrPSc) of normal human prion protein (PrPC) coded by prion gene (PRNP)	[47,48,74,75]
Clinical characteristics of the disease in affected humans	[45,47,48,76]
Disease progression rates	[45,48,64,77,78]
Incubation times in Tg mice expressing homologous human prion protein or its chimera	[52,79,80,81]
Unique neuropathological phenotypes and anatomical distributions of pathogenic PrPSc in the brain	[45,70,82]
Distinct N-linked glycosylation profiles of human PrPSc	[83]
Differential susceptibility of different human prion strains to proteases	[73,84,85,86];
Unique structural organizations of pathogenic prion protein (PrPSc)	[70,76,84,85,86]

## Data Availability

Not applicable.

## Abbreviations

AD	Alzheimer’s disease
CDI	Conformation-Dependent Immunoassay
CJD	Creutzfeldt–Jakob disease
CSA	Conformational Stability Assay
FFI	fatal familial insomnia
FTLD	frontotemporal lobar degeneration
GSS	Gerstmann–Sträussler–Scheinker syndrome
MAPT	microtubule-associated protein tau gene
PrP	prion protein
PrPC	normal or cellular prion protein
PrPSc	pathogenic prion protein
PRNP	prion protein gene
CJD	sporadic Creutzfeldt–Jakob disease
RT QuIC	Real-Time Quaking-Induced Conversion
SFI	sporadic fatal insomnia
VPSPr	variable protease-sensitive prionopathy
